# HLA-DR3 antigen in the resistance to idiopathic dilated cardiomyopathy

**DOI:** 10.1590/1414-431X20165131

**Published:** 2016-03-18

**Authors:** B. Jin, B.W. Wu, Z.C. Wen, H.M. Shi, J. Zhu

**Affiliations:** Department of Cardiology, Huashan Hospital, Fudan University, Shanghai, China

**Keywords:** HLA-DR, Dilated cardiomyopathy, Polymorphism, Meta-analysis

## Abstract

Idiopathic dilated cardiomyopathy (IDC) has been hypothesized as a multifactorial disorder initiated by an environment trigger in individuals with predisposing human leukocyte antigen (HLA) alleles. Published data on the association between HLA-DR3 antigen and IDC risk are inconclusive. To derive a more precise estimation of the relationship, a meta-analysis was performed. Studies were identified by searching the PUBMED and Embase database (starting from June 2015). A total of 19 case-control studies including 1378 cases and 10383 controls provided data on the association between HLA-DR3 antigen and genetic susceptibility to IDC. Overall, significantly decreased frequency of HLA-DR3 allele (OR=0.72; 95%CI=0.58-0.90; P=0.004) was found in patients with IDC compared with controls. When stratified by myocardial biopsy or non-biopsy cases, statistically decreased risk was found for IDC in myocardial biopsy cases (OR=0.69; 95%CI=0.57-0.84; P=0.0003). In the subgroup analysis by ethnicity, borderline statistically significantly decreased risk was found among Europeans from 12 case-control studies (OR=0.76; 95%CI=0.58-1.00; P=0.05). In conclusion, our results suggest that individuals with HLA-DR3 antigen may have a protective effect against IDC.

## Introduction

Idiopathic dilated cardiomyopathy (IDC) is the third most common cause of heart failure characterized by ventricular dilatation and impaired myocardial contractility (1). This multifactorial disease, although closely related to viral and immunologic mechanisms, is also influenced by the complex pattern of inheritance (2). New genetic markers for identifying high-risk populations as well as novel strategies for early detection and preventive care are urgently needed.

In the past decades, a growing number of studies suggested that human leukocyte antigen (HLA)-DR was emerging as a low-penetrant risk factor in the development of IDC. However, the association between HLA-DR3 and IDC risk has been less well characterized. Therefore, this study aimed to derive a more precise estimation of this association by subgroup analysis.

## Material and Methods

### Study search strategy

Case-control studies were identified from PubMed and Embase database in June 2015 using both electronic and manual search strategies. We combined search terms “HLA-DR”, “polymorphism”, and “dilated cardiomyopathy”, and we restricted our search to human populations. When more than one study from the same patient population was published, only the most recent or complete study was selected for this meta-analysis.

### Inclusion criteria

We identified eligible articles on the basis of 3 inclusion criteria:*1* ) case-control studies, *2* ) evaluation of HLA-DR3 antigen in IDC risk, and *3* ) sufficient published data for estimating an odds ratio (OR) with 95% confidence interval (CI).

### Data extraction

Two reviewers (B.J. and B.W.W.) independently extracted data from all selected studies fulfilling inclusion criteria. Disagreement was resolved by discussion between the two reviewers. If these two authors could not reach a consensus, another author (Z.C.W.) was consulted to resolve the dispute and a final decision was made by voting. Data extraction included the first author's surname, publication date, region of origin, ethnicity, myocardial biopsy, source of controls, and demographic data. Data that were not provided in tabular form or in the main text were obtained from online data appendix or from supplementary material.

### Statistical analysis

The Cochrane Collaboration meta-analysis methodology was used for this study. Crude ORs with 95%CIs were used to assess the strength of association between HLA-DR3 antigen and IDC risk. The presence of heterogeneity across trials was evaluated, and P≤0.10 was considered to be significant for statistical heterogeneity. All statistical tests were performed with RevMan version 4.2.2 available free from the Cochrane Collaboration website (http://www.cochrane.org/cochrane/hbook/htm).

## Results

### Study characteristics

A total of 19 case-control studies including 1378 cases and 10383 controls met the inclusion criteria (3 4 5 6 7 8 9 10 11 12 13 14 15 16 17 18 19 20 21). Table 1 lists the eligible studies and their main characteristics. Of the 19 studies, sample sizes ranged from 117 to 2703. Controls were mainly healthy populations and matched for ethnicity and area. Diagnosis of IDC was primarily based on the World Health Organization (WHO) criteria by a panel of clinical cardiologists (22,23).

**Table:**
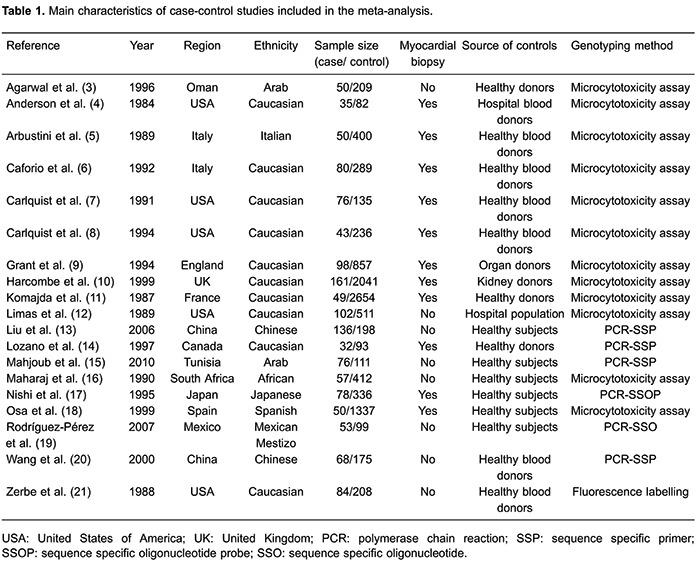

### Main results

**Figure 1:**
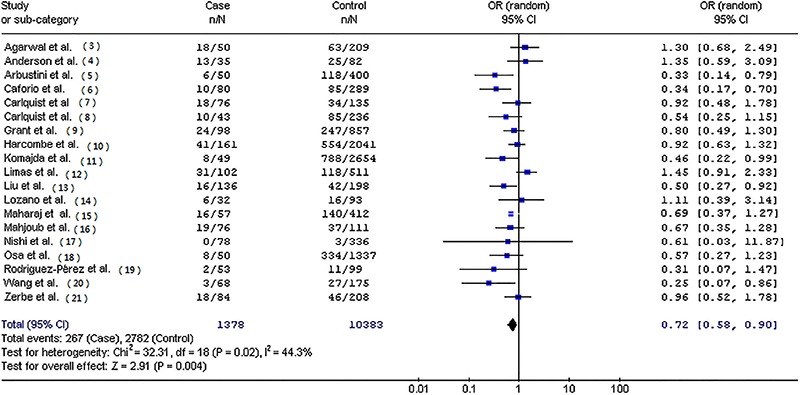
Cumulative odds ratio for human leukocyte antigen-DR3 in patients with idiopathic dilated cardiomyopathy compared with controls from 19 studies (OR=0.72; 95%CI=0.58-0.90; P=0.004).

**Figure 2:**
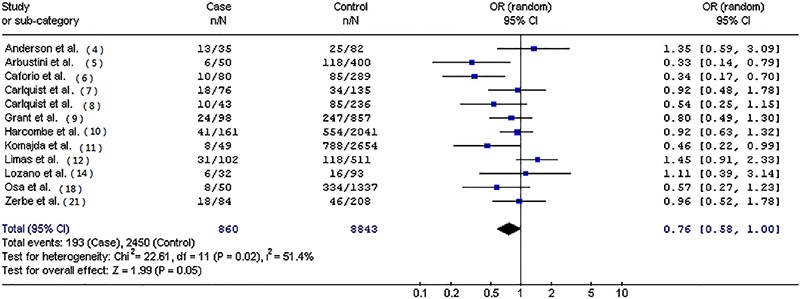
Forest plot of odds ratio for human leukocyte antigen-DR3 in patients with idiopathic dilated cardiomyopathy in a European subset of 12 case-control studies (OR=0.76; 95%CI=0.58-1.00; P=0.05).

Table 2 presents the pooled ORs of this meta-analysis. Overall, significantly decreased frequency of HLA-DR3 allele (OR=0.72; 95%CI=0.58-0.90; P=0.004; Figure 1) was found in patients with IDC compared with controls. When stratified by myocardial biopsy or non-biopsy cases, statistically decreased risk was found for IDC in myocardial biopsy cases (OR=0.69; 95%CI=0.57-0.84; P=0.0003). In the subgroup analysis by ethnicity, significantly decreased risks were found among Europeans (OR=0.76; 95%CI=0.58-1.00; P=0.05; Figure 2) and Asians (OR=0.65; 95%CI=0.46-0.92; P=0.01).

**Table:**
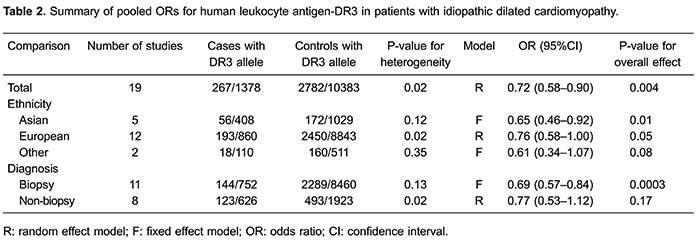

### Sensitivity analysis

Sensitivity analysis was performed by re-running the meta-analysis removing a single study each time to reflect the influence of the individual data-set to the pooled ORs, which were not significantly altered, indicating that our results were statistically robust (24).

### Publication bias

Begg's funnel plot and Egger's test results did not suggest any evidence of publication bias.

## Discussion

The association between a disease susceptibility gene and an HLA subtype may be more apparent than real, especially if multiple loci seem linked to a disease gene (25). It is believed that abnormalities in immunity have been implicated in the pathogenesis of IDC. Certain HLA alleles are associated with the development of particular autoimmune diseases. HLA-DR3 is often associated with an increased risk in non-organ specific autoimmune disease, whereas HLA-DR4 is more frequently associated with organ-specific autoimmune disease. IDC has been hypothesized as a multifactorial disorder initiated by an environmental trigger in individuals with predisposing HLA alleles (26). Published data on the association between HLA-DR3 polymorphism and IDC risk are inconclusive.

The present meta-analysis, including 1378 cases and 10,383 controls from 19 eligible studies, explored the association between the frequency-distribution of HLA-DR3 and IDC risk. Our results indicated that individuals with HLA-DR3 antigen may have a protective effect against IDC. When stratified by myocardial biopsy or non-biopsy cases, statistically decreased risk was found for IDC in myocardial biopsy cases. This finding may be biologically plausible; immune responses are mediated through products of genes located in the major histocompatibility complex (MHC) region, which have the task of presenting cell-associated antigens for their recognition. As the major subregion within the human MHC region, HLA-DR3 is involved in several autoimmune conditions and disease susceptibility. Published studies support the cell-mediated immune response theory as the myocardial inflammatory pathway in unrelated IDC patients (27). It is plausible to consider that individuals with protective HLA alleles could have decreased cross-reactive peptides and fail to develop the disease.

Statistically significant associations between HLA-DR3 and IDC were found in Europeans and Asians but not among other populations, suggesting a possible role of ethnic differences in genetic background and the environment (28). However, the influence of HLA-DR3 allele might be masked by the presence of other as-yet unidentified causal genes involved in IDC development. In addition, the observed ethnic differences might be due to chance because studies with small sample sizes may have insufficient statistical power to detect a slight effect or may generate a fluctuated risk estimate (29).

The statistical correlation of HLA-DR3 antigen with IDC risk is suggestive but deserves further confirmation. Considering the complex nature of IDC, it seems conceivable that HLA-DR3 antigen has only a minor impact on the pathogenesis of IDC. Our findings are consistent with the hypothesis that unrelated IDC may arise in part from abnormal immune regulation, perhaps in conjunction with an infectious process. Although the full extent of these associations and the specific pathogenic mechanisms are at present unknown, the evidence thus far is sufficiently compelling to warrant further investigation.

Some limitations of this meta-analysis should be acknowledged. Firstly, the controls were not uniformly defined. Although most of the controls were selected mainly from healthy populations, some controls were IDC-free subjects. Therefore, non-differential misclassification bias might have occurred because these studies may have included control subjects who have different risk rates of developing IDC. Secondly, heterogeneity is a potential problem when interpreting the results of all meta-analyses. Thirdly, our results were based on unadjusted estimates, while a more precise analysis would be conducted if individual data were available.

In conclusion, our meta-analysis suggests that individuals with HLA-DR3 antigen may have a protective effect against IDC. However, it is necessary to conduct larger sample studies using standardized and unbiased genotyping methods and well-matched controls to confirm the results (30). Moreover, gene-gene and gene-environment interactions should also be considered in the analysis. Such studies may eventually lead to a better, comprehensive understanding of the association between HLA-DR3 antigen and IDC risk.
